# Age-Adjusted and Clinical Probability Adapted D-Dimer Cutoffs to Rule Out Pulmonary Embolism: A Narrative Review of Clinical Trials

**DOI:** 10.3390/jcm13123441

**Published:** 2024-06-12

**Authors:** Marc Righini, Helia Robert-Ebadi, Grégoire Le Gal

**Affiliations:** 1 Division of Angiology and Hemostasis, Geneva University Hospitals and Faculty of Medicine, 1205 Geneva, Switzerland; helia.robert-ebadi@hcuge.ch; 2 Department of Medicine, Ottawa Hospital Research Institute, University of Ottawa, Ottawa, ON K1H 8L6, Canada; glegal@toh.ca; 3 EA3878, University of Brest, 29200 Brest, France

**Keywords:** diagnosis, pulmonary embolism, D-dimer, age-adjusted D-dimer cutoff, clinical probability adapted D-dimer cutoff

## Abstract

Diagnosis of pulmonary embolism remains a challenge for clinicians as its differential diagnosis is wide. The use of sequential diagnostic strategies based on the assessment of clinical probability, D-dimer measurement, and computed tomography pulmonary angiography have been validated in large prospective outcome studies. D-dimer measurement at a standard cutoff of 500 μg/L has gained wide acceptance to rule out pulmonary embolism in around 20 to 30% of patients with a clinically suspected pulmonary embolism. To improve the efficiency of D-dimer measurement, different ways of selecting a higher, albeit safe cutoff were explored: the age-adjusted D-dimer cutoff and the clinical adapted D-dimer cutoff. While both have been prospectively validated in large studies, some differences do exist. In particular, the prevalence of pulmonary embolism in these different validation studies was very different. Overall, the age-adjusted cutoff seems to be safer and less efficient, while the clinical probability adapted cutoff seems more efficient and less safe. Here, we report the available data regarding these two different ways to increase the diagnostic yield of D-dimer. Also, well beyond the accuracy of these adjusted/adapted cutoffs, some external factors, such as the prevalence of pulmonary embolism in the tested population and the clinical setting, have an important impact of the negative predictive value and on the overall efficiency of these cutoffs. Therefore, we also discuss which cutoff should be used according to the expected prevalence of the disease and according to the clinical setting.

## 1. Introduction

Pulmonary embolism (PE) is a frequently suspected diagnosis in the emergency room (ER) in patients presenting with shortness of breath and/or chest pain without any obvious cause identified. Modern PE diagnosis relies on diagnostic strategies, including sequential evaluation of clinical probability, measurement of plasma D-dimer levels, and, most often, CT pulmonary angiography (CTPA) rather than a standalone test. The initial step is the assessment of pre-test clinical probability, either by gestalt or by validated clinical prediction rules (Wells rule of Geneva score) [1,2,3]. This allows separating patients into different groups of PE prevalence, and thus directly influencing the negative and positive predictive values of the diagnostic tests used in these patients [4].

Plasma D-dimer measurement has been extensively evaluated for the exclusion of PE in outpatients. The diagnostic usefulness of the D-dimers lies in their high sensitivity and hence in their capacity to exclude PE when below a certain cutoff (“negative D-dimer”) without further investigations. Indeed, in patients with anon-high clinical probability (low and intermediate groups in a three-level score or unlikely group in a dichotomic score), a highly sensitive negative D-dimer test safely excludes PE without any additional investigation [4]. Sensitive D-dimer tests include those performed by the ELISA technique (median sensitivity 99%; VIDAS^®^ (bioMérieux, Marcy L’Etoile, France), Stratus^®^ (Siemens, Munich, Germany), AxSYM^®^ (Abbott Diagnostics, Abbott Park, IL, USA) and by quantitative latex methods (median sensitivity 96%; STA Liatest^®^ (Stago, Asnières sur Seine, France), Tinaquant^®^ (Roche Diagnostics, Basel, Switzerland)) [5]. In patients with high clinical probability or likely PE, the negative predictive value of even a highly sensitive D-dimer test may be insufficient to exclude PE. D-dimer measurement is thus not used in these patients.

The specificity of the ELISA and quantitative latex D-dimer tests for venous thromboembolism (VTE) is limited, ranging from 35 to 40%. Indeed, D-dimer levels increase in various clinical situations, such as cancer, post-operative periods, infectious/inflammatory states, pregnancy, and with age, leading to a reduced specificity of the test in elderly patients [6,7]. In other words, the probability of having a negative test result is reduced, and the number of patients needed to test (NNT) to exclude one PE without further investigations is higher. Whereas PE can be ruled out in the presence of non-high clinical probability and a negative D-dimer in one out of three outpatients in the emergency room with suspected PE [8], it can be excluded in only one out of twenty patients > 80 years. As current diagnostic strategies include imaging (most often CTPA) in patients with positive D-dimers, a lack of specificity of D-dimers in the elderly can lead to a high proportion of these patients undergoing CTPA.

## 2. The Age-Adjusted D-Dimer Cutoff

The question of a higher D-dimer cutoff in elderly patients was raised many years ago [7], but studies confirming the potential security of such a strategy by retrospectively applying age-adjusted cutoffs to large prospective cohorts of consecutive patients with suspected VTEs were published between 2010 and 2012 [9,10,11,12,13] and confirmed the safety of using an age-adjusted cutoff on an overall population of several thousands of patients.

A progressive age-adjusted D-dimer cutoff (age × 10 μg/L in patients > 50 years) was retrospectively derived and validated in a sample of 1712 patients with suspected PE [9]. The retrospective validation study showed that the age-adjusted cutoff could increase the number of patients in whom the D-dimer test was considered negative by around 20%, without increasing the proportion of false-negative results when compared to the standard cutoff (<500 μg/L). The increase in the diagnostic yield of the D-dimer was particularly pronounced in patients over 80 years, as the age-adjusted cutoff allowed for an increase in the proportion of “negative” D-dimers from 9% to 21% [9], without any false-negative results.

## 3. The ADJUST-PE Study

This progressive age-adjusted D-dimer cutoff was prospectively validated in the ADJUST-PE study, a large multicenter multinational management outcome study [14]. Consecutive patients who presented to the emergency department with clinically suspected PE were assessed by a sequential diagnostic work-up using clinical probability assessment (by one of the two following scores: simplified Geneva score or the two-level Wells score) [2,3,15], highly sensitive D-dimer measurement (ELISA or immuno-turbidimetric assays), and CTPA. Patients with a D-dimer level below their age-adjusted cutoff did not undergo further investigations and were thus left without anticoagulant treatment and followed-up for a period of 3 months [14].

This study included a total of 3346 patients with suspected PE. The subgroup of particular interest for answering the question raised in the ADJUST-PE study was of course patients having D-dimer levels between 500 μg/L and their age-adjusted cutoff (*n* = 337). None of these patients were lost to follow-up, and 6 patients received therapeutic anticoagulation for another indication than VTE. Of the remaining 331, 7 died and 7 had suspected VTE. Only one of these fourteen events was adjudicated as confirmed VTEs (nonfatal PE). The so-called “failure rate” of the age-adjusted cutoff was thus very low at 1/331 (0.3%; 95% CI 0.1–1.7%).

Increasing the proportion of patients in whom PE can be ruled out based on a clinical probability assessment and D-dimer measurement without further testing is particularly interesting in older patients. Indeed, the higher prevalence of renal failure in this population increases the potential risk of contrast-induced nephropathy related to CTPA or even contraindicates this test, and ventilation/perfusion lung scan (which can be performed in patients with severe renal failure) provides a high proportion of inconclusive results in older patients [6]. Moreover, ruling out PE based on clinical probability and a simple blood test could contribute to reducing the time spent in the emergency department and the costs related to PE diagnostic work-ups. Indeed, a previous study had shown that D-the dimer measurement with a conventional cutoff was highly cost-saving in patients less than 80 years, but not in patients over 80 years. Using an age-adjusted D-dimer cutoff dramatically increases the proportion of patients in whom PE can be ruled out and it has been shown to reduce the costs of PE diagnosis in the emergency department. In the ADJUST-PE study, six different D-dimer assays were used depending on the site of inclusion of patients. Therefore, the number of patients with a negative D-dimer, but a value between 500 and their age-adjusted cutoff, was rather limited for each individual test. 

## 4. The RELAX-PE Study

The RELAX-PE study was a real-life study including 1507 patients, which confirmed the safety of the age-adjusted cutoff [16]. Outpatients with suspected PE in whom PE was excluded by a non-high probability and a negative age-adjusted D-dimer, i.e., D-dimer < 500 µg/L up to 50 years, and D-dimer < (age × 10) µg/L in patients above 50 years, were included and followed for three months. The primary outcome was the rate of adjudicated venous thromboembolic events (VTEs).

The 3-month VTE risk in patients left untreated after a negative work-up was 1/1421 (0.07%, 95% CI 0.01–0.40%) in patients with a D-dimer < 500 µg/L and 0/269 (0.0%; 95% CI 0.0–1.41%) after a D-dimer ≥ 500 μg/L but < (age × 10) µg/L. Using the age-adjusted cutoff substantially increased the proportion of patients in whom PE could be excluded without imaging by 20% in the whole cohort and by 67% in patients 75 years or older. Six different D-dimer tests were used: the VIDAS D-dimer exclusion test (bioMérieux, Marcy L’Etoile, France), the Innovance D-dimer (Siemens, Munich, Germany), the Liatest D-dimer (Stago, Asnières sur Seine, France), the AxSYM D-dimer (Abbott Diagnostics, Abbott Park, IL, USA), the HemosIL DD HS (Instrumentation Laboratory, Lexington, MA, USA), and the DPC Immulite 2000 test (Siemens, Munich, Germany).

## 5. Extending the Kind of D-Dimer Assays That Can Be Used with an Age-Adjusted Cutoff

In the ADJUST-PE study, six different D-dimer assays were used depending on the site of inclusion of patients. Table 1 shows the breakdown of the different tests used and the proportion of patients having negative D-dimer results, separated into D-dimer < 500 μg/L and DD ≥ 500 μg/L but < patient’s age-adjusted cutoff. For some of the D-dimer tests used, the number of patients with a value between 500 and their age-adjusted cutoff was rather limited. Therefore, the next step would be to further validate the safety of the age-adjusted D-dimer cutoff by using frozen samples stored during the ADJUST-PE study. Therefore, some other tests (Innovance D-dimer test^®^ (Siemens) on an Atellica COAG 360 automat, the STA Liatest^®^ (Stago) on a STA R Max automat, and a point-of-care LumiraDx^®^) are currently being evaluated on frozen samples arising from the ADJUST-PE study. The analysis is still ongoing, but it should further extend the number of D-dimer tests that can be used to rule out PE with the age-adjusted cutoff.

## 6. The Clinical Probability Adapted D-Dimer Cutoff

In the first algorithms for PE diagnosis, a D-dimer cutoff set at 500 μg/L allowed to rule out PE in 20–30% of patients without performing CTPA, with an overall failure rate of less than 1%. The age-adjusted cutoff discussed above increases to around 40% the proportion of outpatients in whom PE can be ruled out with a very low failure rate. However, this adjusted cutoff also has limitations. Particularly, it increases the yield of D-dimers only in patients aged 50 years or older, and specifically in those older than 75 years. Therefore, other options were developed by researchers.

## 7. The YEARS Model and the YEARS Study

On the basis of a post hoc derivation and validation study (ref), three items of the original Wells’ clinical decision rule—i.e., clinical signs of deep vein thrombosis, hemoptysis, and whether pulmonary embolism is the most likely diagnosis—were the most predictive for pulmonary embolism [19]. They allowed the use of a differential D-dimer threshold based on the presence of one of these items, without losing sensitivity. Hence, this algorithm involves the simultaneous assessment of only the three above-mentioned items and a D-dimer test threshold of 500 μg/L in the presence and 1000 μg/L in the absence of one of the YEARS items.

This simplified diagnostic strategy was used in the YEARS study [17], which showed a 14% absolute reduction in the use of CTPA imaging in comparison with a conventional strategy, without altering the safety outcome, i.e., the rate of venous thromboembolic events (VTE) at three months, which was 0.61 (95% CI: 0.3–0.96%). An external validation study including 3314 patients reported that 42.9% of patients would have PE excluded without the need for imaging, with an overall failure rate of 1.2% (95% CI: 0.8–1.9%), confirming the safety of this strategy. However, among the 272 patients with no YEARS criteria and a D-dimer < 1000 μg/L but above their age-adjusted D-dimer cutoff, PE was diagnosed in 6.3% of them (CI 3.9–9.8%). Therefore, some caution may be needed in this category of patients.

## 8. Another Clinical Probability Adapted D-Dimer Cutoff: The PEGeD Study

In the PEGeD study [18], a simplified diagnostic strategy was proposed in which a modified Wells score was used along with differential D-dimer cutoff values. Pulmonary embolism was ruled out without further testing in patients with a low clinical probability and a D-dimer less than 1000 μg/L as well as in patients with an intermediate clinical probability and a D-dimer less than 500 μg/L. This algorithm was prospectively evaluated in a multicentric Canadian study and resulted in a 17.6% absolute reduction in the use of CTPA imaging in comparison with a conventional strategy [17], without altering the safety outcome, i.e., the rate of venous thromboembolic events (VTEs) at three months, which was 0.05 (95% CI:0.01–0.3%).

An external validation study of the PEGeD algorithm, including 3302 patients, reported that 1621 (49.0%) of patients would have had PE excluded without the need for imaging. Of these patients, 38 (2.3%; 95% CI 1.7–3.2%) had symptomatic PE at initial testing or during the three-month follow-up. Therefore, this external validation study suggested that the algorithm was safe. Upon further analysis, 36 patients out of the 38 patients in whom PE was ruled out based on a low clinical probability and a D-dimer less than 1000 μg/L had a positive age-adjusted D-dimer. Therefore, the risk of VTEs among the 414 patients with a D-dimer below 1000 μg/L but above the age-adjusted D-dimer cutoff was 36/414 (8.7%; 95% CI 6.4–11.8%), suggesting that some caution might be needed in these patients. Table 1 summarizes the data of studies using age-adjusted and clinical probability adapted D-dimer cutoffs.

## 9. Which Cutoff Should We Choose?

A systematic review and individual-patient data meta-analysis was performed on more than 20,000 patients initially included in 16 prospective studies [20]. Overall, D-dimer levels fell below 500 μg/L in 26% to 30% of cases, and below the higher cutoffs in 41% to 47% of cases. Failure rates (missed PE diagnosis) ranged from 1% with a 500-μg/L cutoff to 2.8% with higher cutoffs. When the age-adjusted D-dimer threshold was used, the predicted failure rate varied between 0.76% (95% CI: 0.5–1.1%) and 1.1% (95% CI: 0.8–1.5%). For strategies applying the D-dimer threshold dependent on pretest probability, the predicted failure rate varied between 1.8% (95% CI: 1.4–2.4%) and 2.8% (95% CI: 2.3% to 3.5%). The predicted overall efficiency (PE considered as excluded) was highest for strategies applying a D-dimer threshold dependent on pretest probability and varied from 41% to 47%. The predicted efficiencies for the strategies using the age-adjusted D-dimer threshold varied between 32 and 37%. Overall, these data suggest that the age-adjusted cutoff is safer but less efficient than the clinical probability adapted cutoff to rule out PE.

Another systematic review and individual-patient data meta-analysis performed on more than 20,000 patients analyzed the diagnostic performances of these different D-dimer cutoffs across different healthcare settings. The performance of diagnostic strategies varied considerably across different healthcare settings due to the difference in patient characteristics and the prevalence of PE. For example, the proportion of patients reported to have a thromboembolic event during the 3-month follow-up after a negative age-adjusted D-dimer cutoff was 0.47% (95% CI: 0.18–1.23%) in primary healthcare, 0.65% (95% CI: 0.43–0.99%) in referred secondary care, and 1.7% (95% CI: 0.65–4.25%) in hospitalized patients or nursing home care. The figures with a negative clinical adapted D-dimer cutoff were 0.4 (95% CI: 0.16–1.19%) in primary healthcare, 3.0% (95% CI: 2.47–3.78%) in referred secondary care, and 4.1% (95% CI: 2.54–6.61%) in hospitalized patients or nursing home care.

Regarding efficiency, i.e., the proportion of patients in whom PE could be safely ruled by the clinical probability assessment and D-dimer was as follows: in primary care, 43.5% (95% CI: 29.14–59.03%) for the age-adjusted cutoff and 61.7% (95% CI: 8.33–73.62%) for the clinical probability adapted cutoff; in referred secondary care, 30.46% (95% CI:26.75–34.44%) for the age-adjusted cutoff and 48.75% (95% CI: 43.64–53.94%) for the clinical probability adapted cutoff; in hospitalized patients or nursing home care, 14.8% (95% CI:11.66–18.79%) for the age-adjusted cutoff and 19.4% (95% CI: 15.58–23.99%) for the clinical probability adapted cutoff. Overall, these figures confirm that the safety and diagnostic yield vary according to the clinical settings.

## 10. Conclusions

Both the age-adjusted D-dimer cutoff and the clinical probability adapted cutoff were validated in robust prospective outcome studies. However, some differences exist regarding safety and the diagnostic yield. The age-adjusted cutoff is safer and less efficient; the clinical adapted cutoff is less safe but more efficient. The presented data should help clinicians to balance the trade-off between missing PE cases and decreasing unnecessary CTPA. While the expected prevalence of PE is not always known, it has also an important impact on the safety and efficacy of our diagnostic strategies. Overall, as the age-adjusted cutoff is safer, it seems wise to use it in subgroups of patients with a prevalence higher than 15% or in subgroups of patients at a high risk of PE.

## Figures and Tables

**Table 1:** Main results of studies using age-adjusted or clinically adapted D-dimer cutoffs.

D-Dimer Cutoff	Description of the D-Dimer Use in the Diagnostic Strategy	PE Prevalence	Percentage of CTPA Avoided When Compared with Usual Cutoff	3-Mo TE Rate
Conventional cutoff (500 μg/L) [2,3,8]	D-dimer cutoff: 500 μg/L PE ruled out if negative D-dimer in patients with a low/intermediate or unlikely PTP	20%	-	0.1 (0.0–0.7)
Age-adjusted cutoff (ADJUST PE study) [14]	D-dimer cutoff: age × 10 in patients aged 50 or older, normal cutoff in patients less than 50 years. PE ruled out if negative D-dimer in patients with a low/intermediate or unlikely PTP	19%	12%	0.3 (0.1–1.7)
Age-adjusted cutoff (RELAX-PE study) [16]	D-dimer cutoff: age × 10 in patients aged 50 or older, normal cutoff in patients less than 50 years. PE ruled out if negative D-dimer in patients with a low/intermediate PTP	NA	20%	0.07% (95% CI: 0.01–0.4)
Clinical probability adapted cutoff (YEARS study) [17]	Three criteria from the Wells score: signs of DVT, hemoptysis, PE most likely diagnosis. D-dimer cutoff: 1000 μg/L if no criteria, 500 μg/L if ≥1 criteria	13%	14%	0.8 (0.4–1.5)
Clinical probability adapted cutoff (PEGeD study) [18]	D-dimer cutoff: 1000 μg/L if Wells 0–4 points, 500 μg/L if Wells 4.5–6 points	7.4%	17.6%	0.05 (0.01–0.3)

Abbreviations: PE: pulmonary embolism; PTP: pre-test probability; 3-Mo TE rate: three-month thromboembolic rate.
